# Structure and Characterization of Crimean-Congo Hemorrhagic Fever Virus GP38

**DOI:** 10.1128/JVI.02005-19

**Published:** 2020-03-31

**Authors:** Akaash K. Mishra, Crystal L. Moyer, Dafna M. Abelson, Daniel J. Deer, Kamel El Omari, Ramona Duman, Leslie Lobel, Julius J. Lutwama, John M. Dye, Armin Wagner, Kartik Chandran, Robert W. Cross, Thomas W. Geisbert, Larry Zeitlin, Zachary A. Bornholdt, Jason S. McLellan

**Affiliations:** a Department of Molecular Biosciences, University of Texas at Austin, Austin, Texas, USA; b Mapp Biopharmaceutical, San Diego, California, USA; c Department of Microbiology and Immunology, University of Texas Medical Branch, Galveston, Texas, USA; d Diamond Light Source, Harwell Science and Innovation Campus, Didcot, Oxfordshire, United Kingdom; e Department of Microbiology, Immunology and Genetics, Faculty of Health Sciences, Ben-Gurion University of the Negev, Beer-Sheva, Israel; f Department of Arbovirology, Emerging and Re-emerging Infection, Uganda Virus Research Institute, Entebbe, Uganda; g US Army Medical Research Institute of Infectious Diseases, Fort Detrick, Maryland, USA; h Department of Microbiology and Immunology, Albert Einstein College of Medicine, Bronx, New York, USA; University of Kentucky College of Medicine

**Keywords:** X-ray crystallography, bunyavirus, nairovirus

## Abstract

Crimean-Congo hemorrhagic fever virus (CCHFV) is the causative agent of the most widespread tick-borne viral infection in humans. CCHFV encodes a secreted glycoprotein (GP38) of unknown function that is the target of a protective antibody. Here, we present the crystal structure of GP38 at a resolution of 2.5 Å, which revealed a novel fold primarily consisting of a 3-helix bundle and a β-sandwich. Sequence alignment and homology modeling showed distant homology between GP38 and the ectodomain of Gn (a structural glycoprotein in CCHFV), suggestive of a gene duplication event. Analysis of convalescent-phase sera showed high titers of GP38 antibodies indicating immunogenicity in humans during natural CCHFV infection. The only protective antibody for CCHFV in an adult mouse model reported to date, 13G8, bound GP38 with subnanomolar affinity and protected against heterologous CCHFV challenge in a STAT1-knockout mouse model. Our data strongly suggest that GP38 should be evaluated as a vaccine antigen and that its structure provides a foundation to investigate functions of this protein in the viral life cycle.

**IMPORTANCE** Crimean-Congo hemorrhagic fever virus (CCHFV) is a priority pathogen that poses a high risk to public health. Due to the high morbidity and mortality rates associated with CCHFV infection, there is an urgent need to develop medical countermeasures for disease prevention and treatment. CCHFV GP38, a secreted glycoprotein of unknown function unique to the *Nairoviridae* family, was recently shown to be the target of a protective antibody against CCHFV. Here, we present the crystal structure of GP38, which revealed a novel fold with distant homology to another CCHFV glycoprotein that is suggestive of a gene duplication event. We also demonstrate that antibody 13G8 protects STAT1-knockout mice against heterologous CCHFV challenge using a clinical isolate from regions where CCHFV is endemic. Collectively, these data advance our understanding of GP38 structure and antigenicity and should facilitate future studies investigating its function.

## Abstract (precis)

Crimean-Congo hemorrhagic fever virus (CCHFV) is a priority pathogen that poses a high risk to public health. Due to the high morbidity and mortality rates associated with CCHFV infection, there is an urgent need to develop medical countermeasures for disease prevention and treatment. CCHFV GP38, a secreted glycoprotein of unknown function unique to the *Nairoviridae* family, was recently shown to be the target of a protective antibody against CCHFV. Here, we present the crystal structure of GP38, which revealed a novel fold with distant homology to another CCHFV glycoprotein that is suggestive of a gene duplication event. We also demonstrate that antibody 13G8 protects STAT1-knockout mice against heterologous CCHFV challenge using a clinical isolate from regions where CCHFV is endemic. Collectively, these data advance our understanding of GP38 structure and antigenicity and should facilitate future studies investigating its function.

## INTRODUCTION

Viruses in the *Orthonairovirus* genus are tick-borne members of the *Nairoviridae* family in the *Bunyavirales* order of negative-strand RNA viruses. Orthonairoviruses belong to more than 14 different species and at least 4 of these species contain viruses which can cause disease in humans (1–4). Of these viruses, Crimean-Congo hemorrhagic fever virus (CCHFV) causes the most life-threatening tick-borne viral disease. The disease presents as a severe form of hemorrhagic fever with a case fatality rate of 10% to 40% (4). CCHFV outbreaks have spanned a wide geographic area ranging from Western and Central Asia, the Middle East, Africa, and Southern Europe (5). Increasing global temperatures, migratory birds, and the international livestock trade have all potentially contributed toward the spread of *Hyalomma* ticks—the primary vector for CCHFV (6, 7). Expanding endemic zones, widespread morbidity, and significant mortality make CCHFV an acute threat to public health.

**FIG 1:**
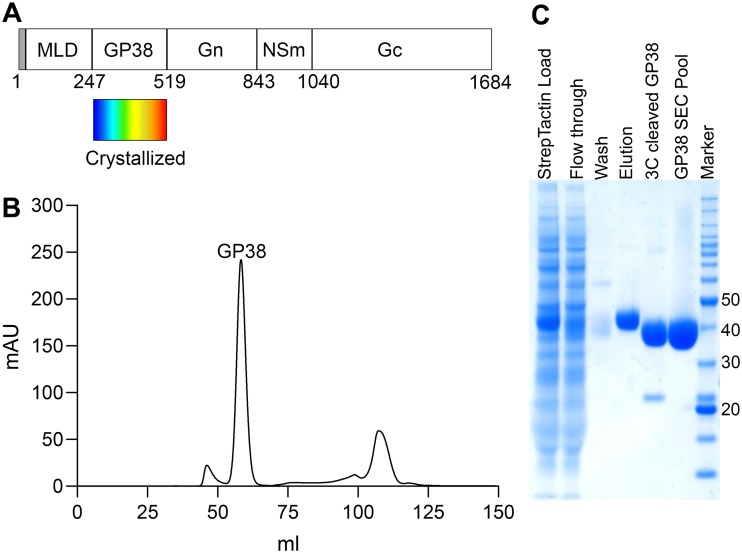
Production of recombinant GP38. (A) Simplified schematic depicting CCHFV GPC encoded by the M segment. The crystallized GP38 portion is highlighted with a rainbow rectangle underneath corresponding to the colors used to depict its structure in Fig. 3. The N-terminal signal peptide is colored in gray. (B) SEC chromatogram of purified GP38 on Superdex 200 column. (C) SDS-PAGE of fractions from GP38 purification. Numbers on the right refer to molecular weight standard (kilodaltons).

Like other nairoviruses, CCHFV has a lipid bilayer envelope and an RNA genome divided into small (S), medium (M), and large (L) segments (8). The CCHFV M segment encodes the glycoprotein precursor complex (GPC) (Fig. 1A), which is cleaved by host-cell proteases into multiple mature proteins. The structural glycoproteins Gn (GPC residues 520 to 842; residue numbering throughout this article is based on CCHFV IbAr 10200) and Gc (GPC residues 1041 to 1684) form spikes on the viral surface and mediate virus entry into target cells, but the role of the secreted glycoproteins remains poorly understood. Nairoviruses are the only bunyaviruses that are known to encode secreted glycoproteins, such as the 38-kDa glycoprotein (GP38) that is produced by CCHFV (9).

GP38 (GPC residues 248 to 519) does not share substantial sequence homology with other viral or cellular proteins. It is *N*-glycosylated in the endoplasmic reticulum (ER) and cleaved by subtilisin kexin isozyme-1/site-1 protease (SKI-1/S1P) in the Golgi complex to liberate it from Gn (10). SKI-1/S1P activity is critical to produce infectious virus from CCHFV-infected cells, indicating that proteolytic processing to separate GP38 from Gn is required for proper virus assembly (11). Later, furin cleavage in the *trans*-Golgi network releases GP38 from the N-terminal mucin-like domain (MLD) (12). Mutating the furin cleavage site abolishes the production of mature GP38 and transiently reduces CCHFV titers but does not abrogate virus replication (13). To date, the function of GP38 is unknown.

Previously, Bertolotti-Ciarlet et al. described murine monoclonal antibodies (MAbs) that provided protection against lethal CCHFV challenge in a neonatal mouse model (14). The neutralizing MAbs were shown to target Gc, whereas the nonneutralizing MAbs were reported to target Gn (14). Golden et al. recently reported that the protective nonneutralizing antibodies, initially thought to target Gn, actually bind to GP38 (15). One of these antibodies, 13G8, was shown to be protective in an adult type-I interferon-deficient mouse model for CCHFV (15). Interestingly, Gc-specific neutralizing MAbs provided no such protection (15). Since no CCHFV Gn-specific MAbs have yet been reported, MAb 13G8 remains the only antibody to show protection against CCHFV in an adult mouse model. This strongly suggests that GP38 plays a role in viral pathogenesis.

To gain insight into the function of GP38, we initiated structural and biophysical studies and obtained a 2.5-Å structure of GP38. GP38 has a novel fold composed primarily of a 3-helix bundle and a β-sandwich. Interestingly, sequence alignment of GP38 showed distant homology with the Gn ectodomain. In addition, the reactivity of convalescent human sera to GP38 indicates it is immunogenic in humans. We also characterized the binding of GP38 to MAb 13G8 and successfully demonstrated the protective efficacy of MAb 13G8 against a heterologous clinical CCHFV isolate strain (Turkey2004) in a STAT1-knockout mouse model. Our data strongly suggest that GP38 should be evaluated as a vaccine antigen.

## RESULTS

### Expression and characterization of CCHFV GP38.

We expressed a construct containing GPC residues 1 to 515 of CCHFV strain IbAr 10200 in FreeStyle 293 cells. The N-terminal MLD (GPC residues 1 to 247) was cleaved at the native furin cleavage site (GPC residues 244 to 247), and secreted GP38 was purified via affinity chromatography followed by size exclusion chromatography (SEC). SEC revealed a sharp symmetrical peak corresponding to a glycosylated GP38 monomer indicating a well-behaved monodispersed protein (Fig. 1B). SDS-PAGE (Fig. 1C) showed a single band at 38 kDa, as has been previously reported for mature GP38 (12). Purification yield from a 500-ml transfection was approximately 4.5 mg of GP38.

**FIG 2:**
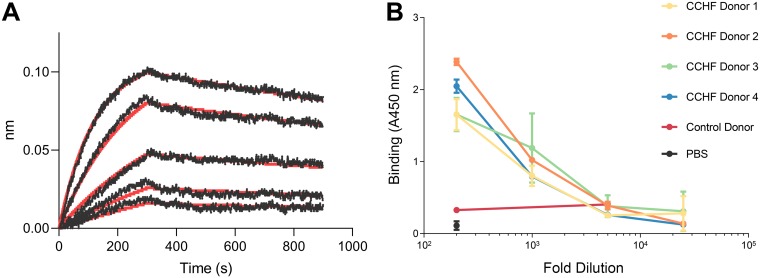
Characterization of GP38 antigen. (A) Binding kinetics of MAb c13G8 and GP38 interaction based on BLI using Octet. Curves were fit to a 1:1 binding model. MAb c13G8 binds GP38 with subnanomolar affinity. (B) GP38 ELISA with serum samples from CCHF convalescent human donors. Serum from all four donors showed similar reactivity toward GP38. Control donor serum or PBS did not show any reactivity toward GP38.

We characterized the recombinant GP38 antigen by evaluating its binding to a chimeric version of MAb 13G8 (denoted c13G8). c13G8 contained the murine variable fragments fused with human constant domains. Biolayer interferometry (BLI) experiments determined that c13G8 bound to GP38 with an equilibrium dissociation constant (*K_d_*) of 0.4 nM, an association rate constant (*k_on_*) of 7.8 × 10^5^ M^−1^s^−1^, and a dissociation rate constant (*k_off_*) of 3.3 × 10^−4^ s^−1^ (Fig. 2A). We also performed an enzyme-linked immunosorbent assay (ELISA) with serum samples from CCHF convalescent human donors in order to test reactivity toward GP38. Serum from four donors showed similar reactivity toward GP38 (Fig. 2B), whereas control donor serum or phosphate-buffered saline (PBS) did not show any reactivity toward GP38. Together, these results indicate that the recombinant GP38 is well folded and that GP38 is immunogenic in humans, consistent with recently reported data (15).

### Crystal structure of GP38.

Crystals of GP38 grew in 0.1 M sodium acetate (pH 5.5), 2% to 4% polyethylene glycol 400 (PEG 400), and 11% to 21% PEG 3350 and belonged to the *P*2_1_ space group. Because GP38 lacks sequence homology to known structures, we collected multiple long-wavelength data sets on the beamline I23 at Diamond Light Source to obtain experimental phases via sulfur single-wavelength anomalous dispersion (SAD). The structure of GP38 was initially determined to 2.8 Å using these data sets, and was subsequently extended to 2.5-Å resolution with an additional data set collected at Advanced Photon Source. The final structure has an *R*_free_ of 25.6% and an *R*_work_ of 22.3% (Table 1).

**TABLE 1:** Data collection, phasing, and refinement statistics for the crystal structure of GP38

Parameter^*a*^	Value(s)^*b*^ for:	
	GP38-APS	GP38-DLS (SAD)
PDB no.	6VKF	
Data collection		
Space group	*P*2_1_	*P*2_1_
Wavelength (Å)	0.979	2.755
Cell dimensions		
*a, b, c* (Å)	62.4, 97.9, 66.0	62.8, 98.0, 66.5
α, β, γ (°)	90, 103.9, 90	90, 103.0, 90
Resolution (Å)	51.48–2.52 (2.63–2.52)	64.85–2.79 (2.94–2.79)
*R*_merge_	0.042 (0.451)	0.083 (2.274)
*I*/σ*I*	12.6 (2.5)	12.0 (1.5)
*CC*_1/2_	0.998 (0.815)	0.999 (0.733)
Completeness (%)	97 (98.2)	95.4 (96)
Redundancy	3.2 (3.3)	15.0 (12.6)
Total reflections	80,537 (9,381)	319,123 (15,337)
Unique reflections	25,110 (2,868)	38,302 (2,584)
Refinement		
Resolution (Å)	51.48–2.52 (2.63–2.52)	
No. of unique reflections	25,070	
*R*_work_/*R*_free_ (%)	22.27/25.58	
No. of atoms	3,901	
Protein	3,793	
Water	10	
NAG	98	
*B*-factors (Å^2^)		
Protein	91.7	
Water	68.4	
NAG	136.2	
RMSDs		
Bond lengths (Å)	0.010	
Bond angles (°)	1.09	
Ramachandran (%)		
Favored	95.4	
Allowed	4.6	
Outliers	0	

a RMSD, root mean square deviation; NAG, N-acetyl glucosamine.

b Data in parentheses are for the highest resolution shell.

**FIG 3:**
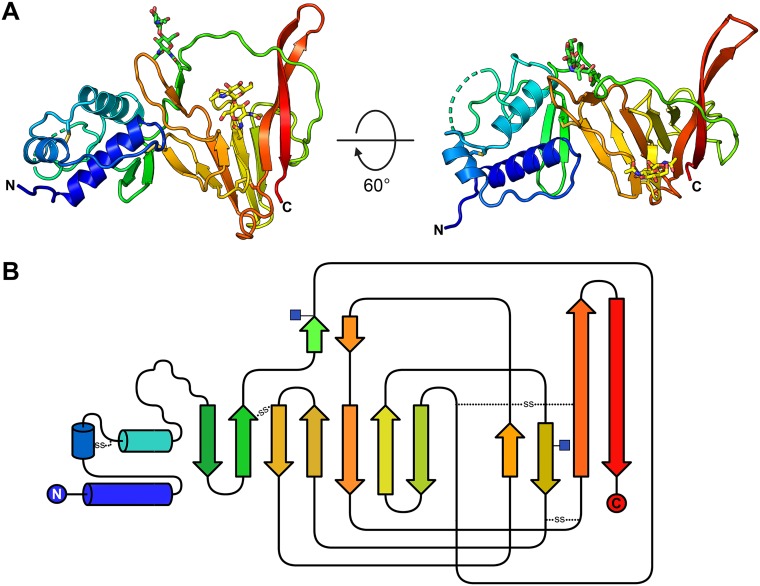
Crystal structure of GP38. (A) GP38 structure is shown in cartoon representation colored as a rainbow from N terminus to C terminus (blue to red). The region between residues 328 and 340 is not well ordered and is represented as a broken dashed line. (B) Topology diagram showing the secondary structure arrangement. The colors of the secondary structure elements match the colors in A. α-Helices and β-strands are represented as cylinders and arrows, respectively. Sites of *N*-linked glycosylation are marked by blue squares. Disulfide bonds are shown by dotted lines.

**FIG 4:**
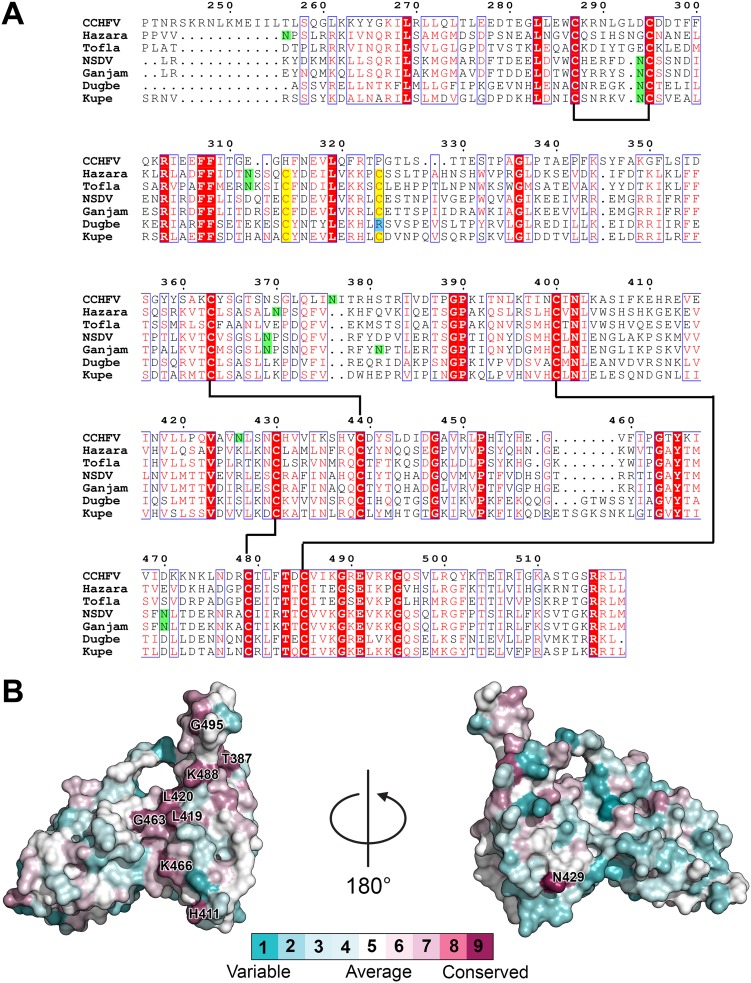
Conservation of GP38 among nairoviruses. (A) Sequence alignment of nairoviruses from CCHFV (CCHFV, Hazara virus, and Tofla virus) and NSDV (NSDV, Ganjam virus, Dugbe virus, and Kupe virus) serogroups. The disulfide bonds in CCHFV GP38 are represented by black lines. The extra cysteine residues in GP38 sequences of other viruses are highlighted in yellow. *N*-Linked glycosylation sites of CCHFV GP38 and putative glycosylation sites in GP38 sequences from other viruses based on NXT/S motif are highlighted in green. Numbering corresponds to CCHFV GP38. (B) ConSurf analysis of GP38 sequences from 13 nairovirus species displayed on the surface of CCHFV GP38 crystal structure. Numbers indicate amino acid residues on CCHFV GP38 at positions that show high conservation scores.

There are two molecules of CCHFV GP38 in the asymmetric unit, denoted chain A and chain B. Chain A has electron density for GPC residues 252 to 322 and 341 to 509, whereas chain B has electron density for GPC residues 250 to 327, 342 to 379, 387 to 486, and 499 to 514. The overall structure of GP38 is defined by a 3-helix bundle at the N terminus followed by a β-sandwich comprising 7- and 4-strand β-sheets (Fig. 3). In addition to the β-sandwich, the β-strand-rich core of GP38 also contains a pair of small antiparallel β-strands (Fig. 3). The region between residues 328 and 340 is disordered, suggesting that this loop is flexible. The CCHFV GP38 fold is stabilized by four disulfide bonds between Cys287 and Cys295, Cys363 and Cys439, Cys400 and Cys485, and Cys430 and Cys479 (Fig. 3 and 4A). The CCHFV GP38 sequence contains *N*-linked glycosylation sequons at Asn376 and Asn426, and both of these sites showed well-resolved electron density for glycans extending away from the protein surface (Fig. 3). No higher order oligomerization was detected from the crystallographic packing, consistent with the SEC data that GP38 is a monomer. As expected from the sequence, a Dali search failed to identify any structural homology to previously determined structures, suggesting a novel three-dimensional (3D) fold (16).

### Conservation of GP38 among nairoviruses.

The N-terminal region of the nairovirus GPC is hypervariable, with large variations in MLD length and different patterns of conserved cysteine residues in the predicted GP38 region. However, all nairoviruses share a SKI-1 cleavage site (R[R/K/H][L/I][L/M]) at the C terminus of GP38, suggesting a release of GP38 from Gn similar to that observed for CCHFV (17). The presence of a furin-like cleavage site (RSKR in CCHFV) between the MLD and GP38 is not clearly apparent in other nairoviruses, although SKI-1-like protease sites have been predicted for many other nairoviruses in the same region (17). Based on sequence alignment, we found that the GP38 sequences of viruses from two different serogroups, namely, the CCHFV serogroup (CCHFV, Hazara virus, and Tofla virus) and the Nairobi sheep disease virus (NSDV) serogroup (NSDV, Ganjam virus, Dugbe virus, and Kupe virus), were strikingly similar (Fig. 4A). The cysteines forming the four disulfide bonds in CCHFV GP38 were conserved across all viruses in these two serogroups, indicating a similar protein fold. An extra pair of cysteines potentially forming an additional disulfide bond was observed for most other viruses in the two serogroups. However, the *N*-linked glycosylation sites for CCHFV GP38 did not align with the putative *N*-linked glycosylation sites in any of the GP38 sequences from the other viruses, suggesting they are not critical to the function of GP38. Among the viruses in the two serogroups, NSDV, Ganjam virus, and Dugbe virus have been reported to cause rare incidents of human infection (18, 19), whereas Hazara virus is considered nonpathogenic for humans and has been used as a model for CCHFV (20, 21). We also performed ConSurf analysis based on GP38 sequences from 13 different nairovirus species in order to predict evolutionarily conserved regions on the GP38 surface (Fig. 4B). Asn402 (buried inside the β-sheet folds) was found to be conserved across all 13 nairoviruses. Based on sequence alignment and ConSurf analysis, it appears that the β-strand-rich C-terminal half of GP38 is relatively more conserved across nairovirus species and is, thus, likely to have a functional role.

### Distant homology of GP38 with Gn.

**FIG 5:**
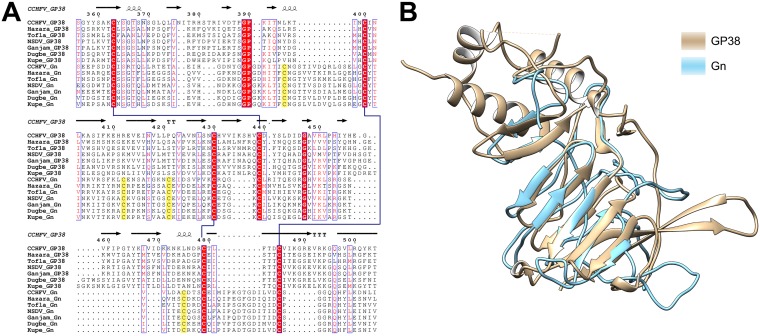
Sequence and structure similarity between CCHFV GP38 and Gn. (A) Sequence alignment of GP38 and Gn ectodomain sequences from CCHFV (CCHFV, Hazara virus, and Tofla virus) and NSDV (NSDV, Ganjam virus, Dugbe virus, and Kupe virus) serogroups. The disulfide bonds in CCHFV GP38 are represented by blue lines. The extra cysteine residues in Gn sequences are highlighted in yellow. Secondary structure elements are displayed based on GP38 structure. Numbering corresponds to CCHFV GP38. (B) Homology model of CCHFV Gn using GP38 structure as the template in Chimera using MODELLER. Gn ectodomain shows homology with the seven-strand β-sheet of GP38.

Sequence alignment of CCHFV GP38 and the Gn ectodomain displayed less than 20% identity. However, alignment of GP38 and Gn ectodomain sequences from CCHFV, Hazara virus, Tofla virus, NSDV, Ganjam virus, Dugbe virus, and Kupe virus revealed that some sequence and structural elements were conserved in the region corresponding to the C-terminal β-strands of GP38 (Fig. 5A). This was also confirmed with secondary structure prediction of the Gn ectodomain using the JPred server, where both GP38 and Gn ectodomain sequences gave the same hits for secondary structure homology (22). Of note, at least three disulfide bonds are conserved between the two glycoproteins (Fig. 5A). Based on these results, we performed homology modeling of the Gn ectodomain using our GP38 structure as a template. The best homology model of the Gn ectodomain using MODELLER shows homology with the seven-strand β-sheet of GP38 (Fig. 5B). This was consistent with our sequence alignment results and secondary structure prediction of Gn using JPred. This distant homology between GP38 and Gn suggests that GP38 may have arisen in nairoviruses due to a gene duplication of Gn or a fragment thereof.

### Protective efficacy of a GP38-directed antibody.

It was previously shown that the murine MAb 13G8 (m13G8) is a nonneutralizing but protective CCHFV antibody, now known to specifically target GP38 (15). m13G8 was raised against strain IbAr 10200 (clade III CCHFV) and was 90% protective against the same strain when administered before virus exposure in adult type I interferon receptor-knockout (IFNAR^−/−^) mice (15). However, the same study showed that m13G8 was only 20% protective against the strain Afg09-2990 (clade IV CCHFV). Here, we extend the evaluation of the protective efficacy of MAb 13G8 in STAT-1^−/−^ mice challenged with Turkey2004, a clade V CCHF virus of great public health importance as it is derived from a region with one of the heaviest annual CCHF incidences in the world (23). In order to also evaluate the potential contribution of Fc effector functions to the *in vivo* protective efficacy of 13G8, we compared the original m13G8 (a murine IgG2b antibody) alongside three chimeric human IgG1 variants of m13G8. The first variant c13G8, as described before, contained the murine variable fragments fused with human IgG1 constant domains. The second was an afucosylated variant of c13G8 (c13G8^AF^-N) that is likely to have significantly increased affinity to FcγR receptors and enhanced Fc effector functions (24). The third was an Fc effector function knockout variant of c13G8 (c13G8^LALAPG^-N) (25).

**FIG 6:**
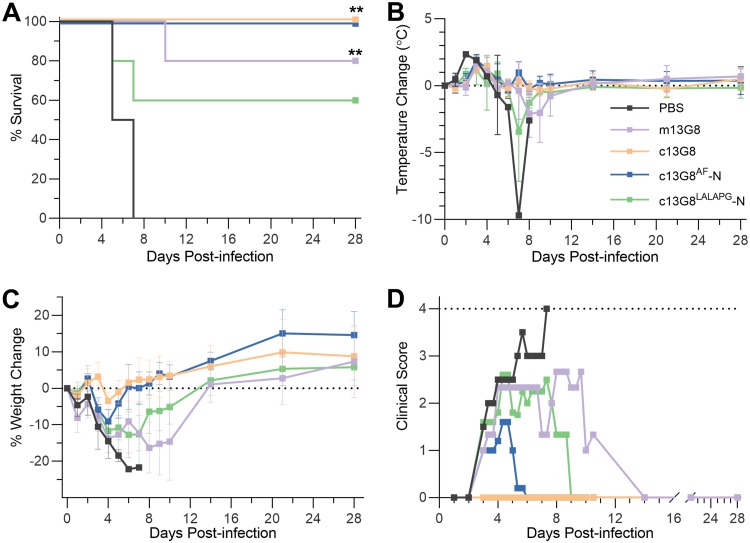
Protection efficacy of MAb 13G8. (A) Survival curves for mice challenged with Turkey2004 and treated with a single 250 μg dose of the indicated MAb 30 min postinfection. **, *P* < 0.01 (Mantel-Cox). (B) Body temperatures taken during the course of the study show the animals registered an elevated temperature by day 4 postinfection. (C) The percent weight change from starting body weight is graphed across the 28-day duration of the experiment. The PBS-, m13G8-, and c13G8^LALAPG^-N-treated groups all display significant levels of weight loss compared to the c13G8- and c13G8^AF^-N-treated groups. (D) Clinical scores of animals within the study cohort are shown. Only animals treated with c13G8 displayed no clinical signs of CCHFV-induced disease for the duration of the experiment. The legend for all the figure panels is displayed in B.

A cohort of 22 mice was divided into 4 treatment groups (*n* = 5/group) and a PBS-treated control group (*n* = 2). All groups received their respective treatments 30 minutes postinfection. The c13G8 and c13G8^AF^-N MAbs both completely protected mice challenged with strain Turkey2004; m13G8 was 80% efficacious and the c13G8^LALAPG^-N MAb showed significant breakthrough, only protecting 60% of the treated animals (Fig. 6A). Temperatures became elevated in all infected animals; however, animals receiving 13G8 treatments displayed a mild and delayed onset of fever compared with the control group (Fig. 6B). Consistent with the survival curves, animals treated with either the m13G8 or c13G8^LALAPG^-N variant displayed severe weight loss and clinical course of disease. In contrast, animals receiving c13G8 showed little to no weight loss and no clinical scores through day 28 (Fig. 6C and D). These results suggest that the Fc region likely contributes to the protective efficacy of 13G8. Of significance, the residual efficacy of c13G8^LALAPG^-N, despite a nonfunctional Fc region, suggests that 13G8 binding to GP38 may also inhibit a currently unknown function of GP38 that contributes to CCHFV virulence.

## DISCUSSION

Despite the severity of CCHFV disease and the increasing number of CCHFV outbreaks, the infectious cycle of this and other nairoviruses remains poorly understood. Nairoviruses are the only bunyaviruses that are known to encode secreted glycoproteins, such as GP38, and the roles of these secreted glycoproteins remain uncharacterized. To express GP38, we used a construct containing the first 515 residues of CCHFV GPC, including the native signal peptide and MLD, which ultimately were removed from mature GP38 by proteolytic processing. However, Golden et al. used a tissue plasminogen activator secretion signal fused to the GP38 coding region to express and purify soluble GP38, indicating that the MLD is not required for proper maturation of GP38 (15). We also report the serum reactivity of convalescent CCHFV donors to GP38 (Fig. 2B). Although most epitope mapping studies from CCHFV-infected human sera have focused on Gn and Gc (26–28), Golden et al. also recently reported GP38 reactivity with convalescent human sera (15). Both our results indicate that GP38 is a natural target of an antibody response in human CCHFV infections.

GP38 is a nairovirus-specific protein with <20% sequence similarity to other viral or cellular proteins. The lack of known GP38 homologs has hindered understanding of its function and contribution to the virus life cycle. Our crystal structure of CCHFV GP38 is a first in the nairovirus family (Fig. 3). We did not identify any similar structures using the Dali search within the PDB, suggesting a novel protein fold. However, our structure provides a 3D template for exploration of GP38 function. Anti-CCHFV antibodies reported by Bertolotti-Ciarlet et al. were characterized by Golden et al. as GP38-directed antibodies and were shown to bind three different epitope regions on GP38 (14, 15). One of these antibodies, 13G8, was found to be protective against CCHFV and remains the only protective antibody reported against CCHFV in an adult mouse model. Scanning mutagenesis and cocrystallization experiments could help identify the epitope for 13G8 and other such protective antibodies identified in future studies.

Using a sequence alignment approach, we discovered that the Gn ectodomain shows homology with the seven strand β-sheet in GP38 and that at least three disulfide bonds are conserved between the two proteins (Fig. 5). This distant homology suggests that GP38 may have arisen via gene duplication of Gn in CCHFV, perhaps explaining why other bunyavirus families lack GP38. Based on our sequence inspection, almost all nairoviruses encode GP38, suggesting that the gene duplication event appears to have occurred early in the evolution of these viruses. Since then, a strong selective advantage must have favored the maintenance of GP38. This is similar to the gene duplication event described for the N-terminal stalk subdomains of Gc in orthobunyaviruses (29). Despite having a low sequence identity (18%), the two stalk subdomains of orthobunyavirus Gc share a fold and play a role in stabilization of the orthobunyavirus particle (29).

The evolutionary advantage of maintaining GP38 within the CCHFV genome is supported by the demonstration that an anti-GP38 MAb (13G8) was able to protect mice from uniformly lethal CCHFV challenge. This strongly suggests that GP38 plays a role in CCHFV virulence. The studies performed here extend the protective range of MAb 13G8 beyond strain IbAr 10200 to include strain Turkey2004 (85% sequence identity to IbAr 10200). The ability of c13G8 to completely protect mice infected with strain Turkey2004 was unexpected given that m13G8 showed only limited protection in mice challenged with strain Afg09-2990 (91% sequence identity to IbAr 10200) (15). In addition, we only used a single dose of 250 μg antibody per mouse for protection compared to two doses of 1 mg antibody per mouse in the previous study. However, it is important to note that STAT-1^−/−^ mice were used in this study, whereas Golden et al. used IFNAR^−/−^ mice for strain IbAr 10200 and strain Afg09-2990 (15).

Golden et al. also examined the role of Fc function and complement fixation in the protective efficacy of m13G8 using various genetically modified mouse models (15). Here, we used a single mouse model to compare the following 13G8 Fc variants: m13G8, c13G8, c13G8^AF^-N, and c13G8^LALAPG^-N. Both c13G8 and c13G8^AF^-N provided complete protection, whereas c13G8^LALAPG^-N protected only 60% of the mice, suggesting that Fc-induced effector functions contributed positively to the protective mechanism of action of 13G8. Moreover, the c13G8^LALAPG^-N variant, having no ability to recruit complement or induce Fc effector functions, still protected STAT-1^−/−^ mice against lethal CCHFV infection. This suggests that MAb 13G8 may also inhibit yet-to-be-determined functions of GP38 that contribute to the pathogenesis of CCFHV. Unfortunately, the vulnerability of MAb 13G8 to GP38 sequence variation across CCHFV clades may limit its utility as a viable immunotherapeutic candidate. Our work calls for further studies to identify broadly reactive and protective GP38 antibodies and to decipher the mechanism of protection by understanding the function of GP38.

## MATERIALS AND METHODS

### Cloning, expression, and purification of CCHFV GP38.

A gene fragment encoding residues 1 to 515 of CCHFV strain IbAr 10200 GPC was codon optimized for human cell expression (via GenScript) and cloned into a pαH eukaryotic expression plasmid with a C-terminal HRV3C protease cleavage site, an 8× HisTag, and a Twin-Strep-tag. This plasmid was transiently transfected into FreeStyle 293 cells (Invitrogen) using polyethyleneimine. Transfected FreeStyle 293 cells were treated with 5 μM kifunensine to ensure uniform high-mannose glycosylation. GP38 was expressed in soluble form and secreted out into the medium, which was harvested and purified over Strep-Tactin resin (IBA Lifesciences). A furin cleavage site (residues 244 to 247) separates the N-terminal MLD and GP38 protein. Because not all of the MLD was cleaved from GP38 by endogenous furin, additional MLD was cleaved off by treatment with furin protease during Strep-tag-based affinity purification. After elution of GP38 from Strep-Tactin resin, the affinity tags were removed by HRV3C cleavage. GP38 was further purified by SEC using a HiLoad 16/600 Superdex 200 pg (GE Healthcare Biosciences) in 2 mM Tris (pH 8.0), 200 mM NaCl, and 0.02% NaN_3_.

### Biolayer interferometry.

A chimeric version of MAb 13G8 (denoted c13G8) was constructed by fusing the variable heavy and light chain fragments from the original murine MAb 13G8 to the constant domains of a human IgG1. The c13G8 MAb was then used for the binding kinetics experiments described herein. Binding kinetics were performed using BLI with an Octet RED96e system (Pall ForteBio). Anti-human IgG Fc Capture (AHC) sensors (Pall ForteBio) were incubated in kinetics buffer (10 mM HEPES, 150 mM NaCl, 3 mM EDTA, 1 mg/ml bovine serum albumin [BSA], and 0.05% Tween 20 [pH 7.5]) for 15 min. Baseline readings were determined by equilibrating sensors for 60 s in the kinetics buffer. MAb c13G8 was diluted in kinetics buffer to a concentration of 5 nM. The AHC sensors were loaded by dipping them into 5 nM MAb c13G8 for a capture time of 150 s. Postcapture baseline readings were determined. The sensors loaded with MAb 13G8 were then dipped into wells with different concentrations of GP38 (0.63 to 10 nM range prepared in kinetics buffer) for a 5-min association, followed by a 10-min dissociation step in the kinetics buffer. A c13G8-captured AHC sensor was also dipped in a well containing kinetics buffer (no GP38) to allow single-reference subtraction to compensate for the slow dissociation of c13G8 MAb from the sensors. The data from AHC sensors were analyzed using the binding kinetics function of the Octet analysis software and fitted to a 1:1 binding model to determine *K_d_*, *k_on_*, and *k_off_*.

### Patient recruitment and ethics statement.

CCHF convalescent patients were recruited with the help of the Uganda Virus Research Institute, Entebbe, Uganda. All survivors had documented clinical history of CCHF infection in Agago and Nakaseke districts, Uganda, ranging from 2013 to 2017. The study was approved by the Helsinki committees of Uganda Virus Research Institute (UVRI), Entebbe, Uganda (reference number GC/127/13/01/15); Soroka Hospital, Beer Sheva, Israel (protocol number 0263-13-SOR); and the Ugandan National Council for Science and Technology (UNCST) (registration number HS1332). Written informed consent, as well as a personal health questionnaire, was completed for each subject who participated in this study. Study participants were all adults and not related. We confirm that all experiments were performed in accordance with the relevant guidelines and regulations.

### GP38 serum reactivity ELISA.

The GP38 serum reactivity ELISA was performed in a Greiner high-binding half-area plate. Individual wells were coated with 250 ng of purified, recombinant GP38 diluted in phosphate-buffered saline (PBS) (pH 7.4). Wells were washed three times with PBS supplemented with 0.05% Tween 20 (PBST) and then blocked with 2% bovine serum albumin (BSA) in PBS. Five-fold serial dilutions of CCHF patient serum, control serum, or PBS were added to the blocked wells and incubated for 1 h at ambient temperature. Samples were prepared in duplicate. After 3 washes with PBST, bound GP38-specific antibody was detected with an anti-human Fc secondary conjugated to horseradish peroxidase (HRP). KPL SureBlue TMB microwell peroxidase was used as the HRP substrate, and the reaction was stopped with an equal volume of 2 N sulfuric acid. The absorbance at 450 nm was measured with a Perkin Elmer EnVision multimode plate reader.

### Crystallization and data collection.

Crystallization trials for GP38 were set up using the sitting-drop vapor diffusion method. The best diffracting crystals were grown in a solution of 0.1 M sodium acetate (pH 5.5), 2% to 4% PEG 400, and 11% to 21% PEG 3350 via hanging-drop vapor diffusion by mixing 1 μl of GP38 (11.2 mg/ml) with 1 μl of reservoir solution. Crystals were looped directly from the crystallization drop and flash frozen in liquid nitrogen. X-ray diffraction data were collected at the 19-ID beamline (Advanced Photon Source; Argonne National Laboratories) and scaled to 2.50 Å. For sulfur-based phasing, long-wavelength diffraction experiments were performed at the long-wavelength MX beamline I23 at Diamond Light Source (30) at an energy of 4.5 keV (λ = 2.7551 Å). In total, three data sets of 3,600 images of 0.1° increments with different κ angles (κ = 0°, κ = −20°, κ = −30°) with 0.1-s exposure times and 50% transmission were recorded from a single crystal.

### Structure determination, model building, and refinement.

Diffraction images from the I23 beamline were recorded on a PILATUS 12M device (Dectris) and processed using XDS and XSCALE (31). The sulfur substructure was determined with SHELXD (32) from the HKL2MAP suite (33) using a 3.8-Å resolution cutoff assuming cysteines were involved in disulfide bridges. The resulting sulfur positions were used in PHENIX.AUTOSOL (34) for phasing. Subsequent automatic model building was carried out with PHENIX.PHASE AND BUILD and BUCCANEER (35). This initial model was completed in COOT (36) to 2.8-Å resolution. The diffraction data from the 19-ID beamline were processed using the CCP4 software suite (37), indexed and integrated in iMOSFLM (38), and scaled and merged with AIMLESS (39). The structure was built manually in COOT using the initial model built above by sulfur phasing. The final structure was refined using PHENIX (40) to an *R*_work_/*R*_free_ of 22.3%/25.6% (Table 1). The structure of GP38 was displayed using PYMOL (41).

### Sequence alignment of GP38 and Gn from nairovirus serogroups.

Clustal Omega was used for sequence alignment of nairovirus GP38 and Gn sequences (42). GP38 and Gn sequences from the following viruses were used for alignment: CCHFV (Q8JSZ3), Hazara virus (A6XIP3), Tofla virus (A0A0U5AG15), NSDV (A0A0A7H8I1), Ganjam virus (A0A191KWA4), Dugbe virus (Q02004), and Kupe virus (B8PWH4). ESPript was used to display the alignment (43).

### ConSurf analysis.

The GP38 crystal structure was used as input along with sequence alignment of GP38 sequences from 13 nairovirus species on the ConSurf Server to calculate evolutionary conservation of amino acid residues (44). Clustal Omega was used to perform sequence alignment (42). To prevent overrepresentation from any particular viral species that has multiple subtypes sequenced, we only included a single amino acid sequence from each viral species. GP38 sequences from the following viruses were used for alignment: CCHFV (UniProt accession number Q8JSZ3), Hazara virus (UniProt accession number A6XIP3), NSDV (UniProt accession number D0PRM8), Dugbe virus (UniProt accession number Q02004), Tamdy virus (UniProt accession number A0A1S5NVL7), Dera Ghazi Khan virus (UniProt accession number A0A191KW89), Artashat orthonairovirus (UniProt accession number A0A1S5NQD0), Hughes orthonairovirus (UniProt accession number A0A191KWA8), Kasokero virus (UniProt accession number A0A0M4LB14), Keterrah virus (UniProt accession number A0A0M4KM64), Qalyub orthonairovirus (UniProt accession number A0A1S5NQX5), Sakhalin orthonairovirus (UniProt accession number A0A1S5NQC0), and Thiafora orthonairovirus (UniProt accession number A0A0M5KMK1). PYMOL was used for visualization of surface conservation (41).

### CCHFV Gn secondary structure analysis and homology modeling.

The JPred server was used for secondary structure prediction of Gn (22). Clustal Omega was used for sequence alignment of CCHFV GP38 and Gn sequences (42). Based on sequence and secondary structure alignment, a homology model of Gn was obtained using the GP38 structure as a template via MODELLER in Chimera (45, 46). The best fit output model was selected as the model for Gn.

### UTMB animal welfare, observation, and euthanasia criterion.

Animal studies were approved by The University of Texas Medical Branch at Galveston (UTMB) Institutional Animal Care and Use Committee (IACUC). Animal research was carried out in compliance with the Animal Welfare Act and other federally regulated stipulations regarding animals and adherences to the Guide for the Care and Use of Laboratory Animals, National Research Council, 2013. The animal facilities where this research was conducted are accredited by the Association for Assessment and Accreditation of Laboratory Animal Care International. Euthanasia criteria was defined as the follows: mouse displays severely hunched posture, inability or reluctance to move, appears weak (staggering when moving around cage), labored breathing, or weight loss of greater than 25% of starting body weight.

### CCHFV challenge stocks.

The Turkey-200406546 strain (referred as Turkey2004, throughout) (kindly provided by T. Ksiazek, UTMB, World Reference Center for Emerging Viruses and Arboviruses, Galveston, TX) was propagated in Vero E6 cells once followed by expansion in SW13 human adenocarcinoma cells. All work with live CCHFV was performed in a biosafety level 4 facility at the Galveston National Laboratory, University of Texas Medical Branch, Galveston, TX. All cell and viral stocks were tested and are free of mycoplasma by using a PCR kit (IntronBio, Gyungg-Do, South Korea).

### Anti-CCHFV antibodies.

m13G8 was obtained from BEI Resources by UTMB. c13G8, c13G8^AF^-N, and c13G8^LALAPG^-N were constructed and purified at Mapp Biopharmaceutical. c13G8 was expressed transiently in expiCHO cells (Thermo Fisher Scientific). c13G8 was purified from expiCHO cell supernatants using a GE MabSelect SuRe LX protein A affinity chromatography column on an AKTA pure fast protein liquid chromatography (FPLC) system. The c13G8 antibody was eluted using a glycine elution buffer at pH 2.2 and neutralized with 2 M Tris base to a pH of ∼7. The c13G8^AF^-N and c13G8^LALAPG^-N afucosylated antibodies were expressed in Nicotiana benthamiana plants (tobacco plants). The plant-derived antibodies were purified as described previously (47).

### CCHFV mouse challenge studies.

The study utilized 4- to 8-week-old female 129S6/SvEv-*Stat1^tm1Rds^* mice (STAT-1^−/−^) (Taconic, Germantown, NY). After an acclimatization period in barrier conditions within environmentally enriched sterile housing, mice were anesthetized by isoflurane and implanted with subdermal transponders, which provide coded identifiers and permitted body temperature monitoring and measurements (Biomedic Data Solutions, Seaford, DE). Challenge material preparations were diluted in Hanks balanced salts medium with 2% fetal bovine serum (FBS) for a final targeted challenge dose of 100 PFU. All challenge doses were frozen for storage and verified by back-titrations by plaque assay on SW-13-CDC cells (48). After anesthesia by isoflurane, 500 μl of each preparation was administered intraperitoneally (i.p.), with five STAT-1^−/−^ mice per experimental group. Thirty minutes after challenge, all treatment groups were administered approximately 5 mg/kg (250 μg/mouse) of each antibody per treatment group. Animals were then observed for clinical scoring, temperature, and weight change. Mice were monitored daily and euthanized upon reaching institutionally approved endpoint score criteria or study endpoint (28 days postinfection [p.i.]).

### Data availability.

The atomic coordinates and structure factors for CCHFV GP38 have been deposited in the Protein Data Bank (PDB) under accession number 6VKF.
